# Supplementary material for: Trace Element Bioaccumulation in Stone Curlew (Burhinus oedicnemus, Linnaeus, 1758): A Case Study from Sicily (Italy)
Source: Int J Mol Sci. 2020 Jun 28;21(13):4597. doi: 10.3390/ijms21134597 (PMC7370152; doi:10.3390/ijms21134597)
Supplement: Supplementary file 1 [file ijms-21-04597-s001.zip › Table S1.pdf]

Table S1. Limit of detection (LOD) and Limit of Quantification (LOQ) for each metal and matrix (mg/Kg dry weight).

Metals	Blood		Feather		Soil	
mg/Kg dry weight	LOD	LOQ	LOD	LOQ	LOD	LOQ
As	0.001	0.006	0.004	0.016	0.912	3.124
Cd	0.001	0.006	0.001	0.004	0.302	1.127
Co	0.005	0.022	0.039	0.160	0.101	0.409
Cr	0.073	0.154	0.028	0.139	0.904	3.105
Cu	0.006	0.025	0.043	0.186	0.101	0.501
Hg	0.003	0.011	0.009	0.037	0.010	0.022
Mn	0.033	0.145	0.099	0.412	0.200	0.504
Ni	0.050	0.265	0.052	0.225	0.307	0.910
Pb	0.031	0.155	0.006	0.025	0.704	2.011
Zn	0.025	0.241	0.197	0.836	3.148	9.181
Se	0.004	0.017	0.007	0.029	0.125	0.301
V	0.004	0.019	0.183	0.812	1.154	5.121
