# Supplementary material for: Trace Element Bioaccumulation in Stone Curlew (Burhinus oedicnemus, Linnaeus, 1758): A Case Study from Sicily (Italy)
Source: Int J Mol Sci. 2020 Jun 28;21(13):4597. doi: 10.3390/ijms21134597 (PMC7370152; doi:10.3390/ijms21134597)
Supplement: Supplementary file 1 [file ijms-21-04597-s001.zip › Table S2.pdf]

Table S2. Descriptive statistics of the concentrations of bioaccumulated trace elements in abrased back feathers (mg/Kg d.w.).

Penisola Magnisi		As	Cd	Co	Cr	Cu	Hg	Mn	Ni	Pb	Se	V	Zn
N.		32	31	32	30	32	32	32	32	32	32	32	32
Mean		0.180	0.032	0.288	2.860	9.420	0.416	8.564	1.199	0.848	0.483	1.299	60.853
Median		0.152	0.027	0.252	2.219	8.779	0.386	8.474	0.943	0.730	0.506	1.252	58.353
S.D.		0.097	0.025	0.133	2.115	2.942	0.186	2.968	0.806	0.409	0.192	0.435	16.035
Min.		0.055	0.009	0.109	0.788	5.657	0.175	4.146	0.482	0.279	<0.007	0.524	37.743
Max.		0.478	0.136	0.609	10.232	18.256	0.792	15.653	4.622	1.998	0.908	2.463	109.084
Percentile	25	0.112	0.016	0.199	1.198	7.250	0.255	6.384	0.811	0.524	0.375	1.061	51.122
	50	0.152	0.027	0.252	2.219	8.779	0.386	8.474	0.943	0.730	0.506	1.252	58.353
	75	0.211	0.039	0.314	3.842	10.400	0.576	9.639	1.265	1.059	0.584	1.486	64.858
Ragusa		As	Cd	Co	Cr	Cu	Hg	Mn	Ni	Pb	Se	V	Zn
N.		31	28	31	29	31	31	31	31	31	31	31	31
Mean		0.139	0.029	0.303	2.742	9.007	0.376	10.060	1.266	0.497	0.566	1.352	62.085
Median		0.137	0.022	0.306	2.021	8.980	0.389	10.314	1.122	0.456	0.594	1.336	60.012
S.D.		0.054	0.020	0.115	1.900	1.991	0.135	3.608	0.722	0.190	0.224	0.469	10.370
Min.		0.025	0.007	0.088	0.713	4.594	0.152	2.963	0.192	0.131	0.129	0.184	42.812
Max.		0.286	0.084	0.635	8.274	15.856	0.713	16.522	3.300	0.955	0.930	2.599	87.525
Percentile	25	0.100	0.014	0.224	1.461	7.761	0.256	6.548	0.856	0.360	0.346	1.068	54.558
	50	0.137	0.022	0.306	2.021	8.980	0.389	10.314	1.122	0.456	0.594	1.336	60.012
	75	0.163	0.036	0.376	3.729	9.963	0.447	12.393	1.702	0.652	0.672	1.595	70.207
Piana d Gela		As	Cd	Co	Cr	Cu	Hg	Mn	Ni	Pb	Se	V	Zn
N.		30	30	30	29	30	30	30	30	30	30	30	30
Mean		0.285	0.010	0.393	2.452	10.776	0.193	17.238	1.554	0.458	0.802	2.606	72.555
Median		0.277	0.009	0.385	2.343	10.783	0.177	16.630	1.470	0.453	0.778	2.450	71.397
S.D.		0.100	0.004	0.131	1.031	1.880	0.095	5.987	0.711	0.110	0.217	0.924	14.349
Min.		0.155	0.005	0.192	1.140	7.562	0.024	8.126	0.748	0.265	0.441	1.362	39.386
Max.		0.534	0.022	0.717	6.211	14.724	0.513	28.224	4.533	0.718	1.269	5.157	127.105
Percentile	25	0.209	0.008	0.289	1.829	9.299	0.142	12.279	1.110	0.378	0.632	1.860	67.486
	50	0.277	0.009	0.385	2.343	10.783	0.177	16.630	1.470	0.453	0.778	2.450	71.397
	75	0.350	0.011	0.462	2.688	11.792	0.223	21.517	1.721	0.533	0.972	3.112	77.018
