# Supplementary material for: Trace Element Bioaccumulation in Stone Curlew (Burhinus oedicnemus, Linnaeus, 1758): A Case Study from Sicily (Italy)
Source: Int J Mol Sci. 2020 Jun 28;21(13):4597. doi: 10.3390/ijms21134597 (PMC7370152; doi:10.3390/ijms21134597)
Supplement: Supplementary file 1 [file ijms-21-04597-s001.zip › Table S3.pdf]

Table S3. Descriptive statistics of the concentrations of bioaccumulated trace elements in blood (mg/kg d.w.).

Penisola Magnisi		As	Cd	Co	Cr	Cu	Hg	Mn	Ni	Pb	Se	V	Zn
N.		32	32	32	32	32	31	32	32	32	32	32	32
Mean		0.014	0.002	0.035	0.086	0.813	0.117	0.330	<0.050	0.050	0.804	0.028	7.927
Median		0.012	<0.001	0.029	<0.073	0.796	0.105	0.298	<0.050	<0.031	0.790	0.037	7.935
S.D.		0.013	0.001	0.024	0.052	0.139	0.058	0.110	0.002	0.075	0.260	0.025	0.856
Min.		<0.001	<0.001	0.013	<0.073	0.523	0.053	0.201	<0.050	<0.031	0.152	<0.004	6.591
Max.		0.059	0.005	0.146	0.342	1.196	0.306	0.665	0.059	0.444	1.219	0.070	10.697
Percentile	25	0.005	0.001	0.021	0.073	0.726	0.076	0.245	0.050	0.031	0.612	0.004	7.207
	50	0.012	0.001	0.029	0.073	0.796	0.105	0.298	0.050	0.031	0.790	0.037	7.935
	75	0.017	0.002	0.038	0.073	0.871	0.144	0.398	0.050	0.032	1.008	0.050	8.346
**Ragusa**		**As**	**Cd**	**Co**	**Cr**	**Cu**	**Hg**	**Mn**	**Ni**	**Pb**	**Se**	**V**	**Zn**
N.		28	28	28	27	28	28	28	28	28	28	28	28
Mean		0.010	0.002	0.058	0.076	0.799	0.136	0.355	<0.050	0.058	1.135	0.044	8.326
Median		0.011	0.002	0.051	<0.073	0.799	0.143	0.328	<0.050	<0.031	1.130	0.048	8.161
S.D.		0.007	0.001	0.029	0.018	0.149	0.044	0.136	0.032	0.092	0.341	0.028	0.869
Min.		<0.001	<0.001	0.020	<0.073	0.524	0.055	0.183	<0.050	<0.031	0.357	<0.004	7.240
Max.		0.021	0.005	0.129	0.153	1.146	0.226	0.795	0.213	0.456	1.668	0.093	11.097
Percentile	25	0.002	0.001	0.036	0.073	0.706	0.099	0.259	0.050	0.031	0.927	0.012	7.752
	50	0.011	0.002	0.051	0.073	0.799	0.143	0.328	0.050	0.031	1.130	0.048	8.161
	75	0.015	0.003	0.072	0.073	0.869	0.166	0.401	0.050	0.031	1.393	0.060	8.922
**Piana d Gela**		**As**	**Cd**	**Co**	**Cr**	**Cu**	**Hg**	**Mn**	**Ni**	**Pb**	**Se**	**V**	**Zn**
N.		26	27	27	27	27	27	27	27	27	27	27	27
Mean		0.028	0.002	0.043	0.143	0.915	0.106	0.449	0.067	0.042	1.454	0.065	8.174
Median		0.021	0.002	0.034	0.073	0.910	0.095	0.358	<0.050	0.036	1.348	0.067	7.883
S.D.		0.022	0.001	0.024	0.197	0.076	0.048	0.246	0.037	0.013	0.566	0.009	0.858
Min.		<0.001	<0.001	0.014	0.073	0.747	0.056	0.272	<0.050	<0.031	0.733	0.041	7.203
Max.		0.079	0.004	0.101	1.002	1.071	0.276	1.360	0.238	0.083	3.685	0.082	10.767
Percentile	25	0.009	0.002	0.028	0.073	0.879	0.073	0.326	0.050	0.031	1.102	0.060	7.584
	50	0.021	0.002	0.034	0.073	0.910	0.095	0.358	0.057	0.036	1.348	0.067	7.883
	75	0.047	0.003	0.055	0.073	0.963	0.118	0.461	0.067	0.051	1.585	0.072	8.616
