# Supplementary material for: Trace Element Bioaccumulation in Stone Curlew (Burhinus oedicnemus, Linnaeus, 1758): A Case Study from Sicily (Italy)
Source: Int J Mol Sci. 2020 Jun 28;21(13):4597. doi: 10.3390/ijms21134597 (PMC7370152; doi:10.3390/ijms21134597)
Supplement: Supplementary file 1 [file ijms-21-04597-s001.zip › Table S4.pdf]

Table S4. Descriptive statistics of the concentrations (mg/Kg d.w.) of bioaccumulated trace elements in soil samples, legal fixed by the Legislative Decree No. 152/2006 approving the Code on the Environment.

Penisola Magnisi		As	Cd	Co	Cr	Cu	Hg	Mn	Ni	Pb	Se	V	Zn
N.		5	5	5	5	5	5	5	5	5	5	5	5
Mean		18.435	0.221	4.112	18.890	18.471	0.113	342.801	22.969	33.080	3.078	52.911	49.516
Median		19.158	0.222	3.346	20.600	18.526	0.127	374.502	25.800	30.400	3.071	55.200	63.855
S.D.		3.046	0.053	1.630	6.298	4.806	0.035	88.466	5.168	6.073	0.510	21.096	23.742
Min.		13.482	0.139	2.549	11.400	13.400	0.051	216.535	16.932	29.482	2.370	29.681	18.924
Max.		21.084	0.286	6.275	26.104	24.606	0.132	431.400	27.711	43.800	3.647	77.400	68.600
Percentile	25	15.666	0.179	2.769	12.374	13.772	0.089	252.680	17.366	29.601	2.601	31.541	23.862
	50	19.158	0.222	3.346	20.600	18.526	0.127	374.502	25.800	30.400	3.071	55.200	63.855
	75	20.842	0.262	5.838	24.552	23.143	0.131	417.073	27.156	37.900	3.559	73.138	68.000
Ragusa		As	Cd	Co	Cr	Cu	Hg	Mn	Ni	Pb	Se	V	Zn
N.		5	5	5	5	5	5	5	5	5	5	5	5
Mean		9.607	0.223	10.209	30.238	14.708	0.016	530.500	18.733	21.594	2.485	54.116	33.259
Median		10.236	0.213	9.761	33.068	14.528	0.016	548.000	17.773	19.531	2.412	57.570	30.080
S.D.		4.327	0.074	2.061	5.390	3.191	0.002	191.137	2.742	4.953	0.344	7.421	8.934
Min.		5.098	0.143	8.367	23.800	10.196	0.014	240.637	16.000	15.936	2.136	45.618	25.200
Max.		16.078	0.345	13.189	35.630	18.268	0.019	766.064	22.441	27.400	2.984	61.024	45.472
Percentile	25	5.649	0.171	8.384	24.450	11.798	0.015	368.219	16.367	17.430	2.183	46.109	25.548
	50	10.236	0.213	9.761	33.068	14.528	0.016	548.000	17.773	19.531	2.412	57.570	30.080
	75	13.252	0.280	12.259	34.612	17.709	0.018	684.032	21.579	26.791	2.823	60.395	42.560
Piana d Gela		As	Cd	Co	Cr	Cu	Hg	Mn	Ni	Pb	Se	V	Zn
N.		5	5	5	5	5	5	5	5	5	5	5	5
Mean		20.275	0.063	11.366	11.178	30.076	0.019	643.639	12.260	10.154	2.867	19.027	24.967
Median		19.922	0.064	11.400	11.614	29.420	0.021	646.813	11.373	10.800	3.422	19.020	26.275
S.D.		2.976	0.014	4.130	1.186	17.932	0.007	158.668	3.125	1.624	1.092	4.138	7.551
Min.		17.331	0.049	7.171	9.562	9.960	0.010	493.600	8.465	8.367	1.372	13.745	15.936
Max.		25.197	0.084	16.400	12.549	56.200	0.028	869.685	16.000	11.765	4.021	24.706	33.922
Percentile	25	18.028	0.050	7.286	9.981	13.880	0.012	494.111	9.512	8.416	1.736	15.273	17.319
	50	19.922	0.064	11.400	11.614	29.420	0.021	646.813	11.373	10.800	3.422	19.020	26.275
	75	22.699	0.075	15.429	12.157	46.600	0.025	791.581	15.451	11.569	3.722	22.786	31.961
Italian D.Lgs. 152-2006		20	2	20	150	120	1	-	120	100	3	90	150
